# Supplementary material for: Dynalign II: common secondary structure prediction for RNA homologs with domain insertions
Source: Nucleic Acids Res. 2014 Nov 21;42(22):13939–48. doi: 10.1093/nar/gku1172 (PMC4267632; doi:10.1093/nar/gku1172)
Supplement: SUPPLEMENTARY DATA [file supp_gku1172_nar-02021-z-2014-File012.zip › manual/javadoc/RNAstructure_java_drawing/source/menus/package-summary.html]

RNAstructure\_java\_drawing.source.menus


JavaScript is disabled on your browser.


- Overview
- Package
- Class
- Tree
- Index
- Help

- Prev Package
- Next Package

- Frames
- No Frames

- All Classes

# Package RNAstructure\_java\_drawing.source.menus

A package that holds all menus used in the drawing framework.

See: Description

- Class Summary

  | Class | Description |
  | --- | --- |
  |  |  |
  | --- | --- |
  | AnnotationMenu | A class that handles a menu which can annotate a drawn structure. |
  | DotsMenu | A class that handles a menu which can manipulate a drawn dot plot. |
  | PlotMenu | A class that handles a menu which can manipulate a drawn dot plot. |
  | StructureMenu | A class that handles a menu which can manipulate a drawn structure. |


## Package RNAstructure\_java\_drawing.source.menus Description

A package that holds all menus used in the drawing framework.

- Overview
- Package
- Class
- Tree
- Index
- Help

- Prev Package
- Next Package

- Frames
- No Frames

- All Classes
